# Dr. Lohmeyer on the Results of Revaccination in the Prussian Army, in the Year 1840

**Published:** 1842-06-04

**Authors:** 


***Results of Revaccination in the Prussian Army, in the Year* 1840.** By Dr. Lohmeyer.—In the last year there were vaccinated in all the regiments together, 43,522 individuals. On these the cicatrices from previous vaccinations were

| | | |
|---|---|---|
| | distinct in . . . . | 34573 |
| | indistinct in . . . | 6177 |
| | not discernible in . | 2772 |

The pustules produced by the present vaccinations were

| | |
|---|---|
| regular in their course in . . . . | 20952 |
| and irregular in . . . . . . . | 8820 |
| and no effect at all was produced in . | 13750 |

The vaccinations that had thus taken no effect were repeated,

| | |
|---|---|
| with success in . . | 2831 |
| without success in . | 8958 |

The numbers of genuine pustules produced were as follows:

| | | |
|---|---|---|
| 1 to 5 | in | 10021 |
| 6 to 10 | in | 5875 |
| 11 to 20 | in | 4171 |
| 21 to 30 | in | 885 |

Of those who had been successfully vaccinated in 1840, and previously, there were attacked during the past year,

| | |
|---|---|
| with varicella . . | 7 |
| with varioloid . . | 2 |
| with true variola . | 1 |

In the past year more frequently than in any other, the vaccination was performed with lymph taken from genuine and regular vaccine pustules on adult persons; for past experience has consequently led military practitioners to be more strongly convinced that for the vaccination of adults, lymph taken from adults is to be preferred to that taken from children, and that it produces a more powerful reaction, and more strongly-developed pustules. And that the pustules thus produced do actually protect from the contagion of small-pox is proved by this, that in the 4th corps, in which the plan has been long followed of vaccinating adults with lymph taken from good pustules in other adults, there has not occurred, during three years, a single case of any variety of small-pox among those in whom the vaccine pustule ran its ordinary course, nor more than one case among those who were unsuccessfully revaccinated, although small-pox was more or less rife in the neighbourhood in which the corps was quartered.

The results of past years have shown a regularly increasing ratio in the proportion of successful vaccinations to the number altogether vaccinated; and this increase has continued in the year 1840, for of 43,522 revaccinated, 20,952 have exhibited genuine and regular pustules. Of all the revaccinations, therefore, 48 per cent. were successful; while in 1839, the proportion was only 46, in 1838 and 1837 only 45; in 1836, 43; in 1835, 39; in 1834, 37; and in 1833, only 31 per cent. The proportions varied in 1840 between 40 per cent., and 60 per cent., in different regiments.

With respect to the advantage of revaccination as a preventive of small-pox, the experience of 1840 proves it in a striking manner; for during that year there occurred only 74 cases in the whole army, and of these 46 were cases of varicella, 21 of varioloid disease, and only 7 of genuine small-pox. Of the last two proved fatal; and neither of the patients had been revaccinated after his enlistment. In general, just as in former years, most of the cases of spurious and genuine small-pox occurred in recruits soon after their enlistment, and before they had been revaccinated. Of those who had been revaccinated it has been already stated that only ten were affected, and of these, 7 had varicella, 2 varioloid disease, and only one a genuine variola.—*British and Foreign Med. Rev., from Medicinische Zeitung.* April 21, 1841.

